# Phytosynthesis of Biological Active Silver Nanoparticles Using *Echinacea purpurea* L. Extracts

**DOI:** 10.3390/ma15207327

**Published:** 2022-10-20

**Authors:** Ioana Catalina Fierascu, Irina Fierascu, Anda Maria Baroi, Camelia Ungureanu, Alina Ortan, Sorin Marius Avramescu, Raluca Somoghi, Radu Claudiu Fierascu, Cristina Elena Dinu-Parvu

**Affiliations:** 1 Faculty of Pharmacy, University of Medicine and Pharmacy “Carol Davila”, 37 Dionisie Lupu Str., 030167 Bucharest, Romania; 2 National Institute for Research & Development in Chemistry and Petrochemistry—ICECHIM Bucharest, 202 Spl. Independentei, 060021 Bucharest, Romania; 3 Faculty of Horticulture, University of Agronomic Sciences and Veterinary Medicine of Bucharest, 011464 Bucharest, Romania; 4 Faculty of Chemical Engineering and Biotechnologies, University “Politehnica” of Bucharest, 313 Splaiul Independentei Str., 060042 Bucharest, Romania; 5 Research Center for Environmental Protection and Waste Management (PROTMED), University of Bucharest, 91–95 Splaiul Independentei, 050095 Bucharest, Romania

**Keywords:** *Echinacea purpurea* L., natural extracts, phytosynthesis, silver nanoparticles, antioxidant properties, antimicrobial activity

## Abstract

With their phytoconstituents acting as reducing and capping agents, natural extracts can be considered a viable alternative for the obtaining of metallic nanoparticles. The properties of phytosynthesized nanoparticles are dependent upon size and morphology, which, in turn, can be tailored by adjusting different parameters of the phytosynthesis process (such as the extracts’ composition). In the present study, we aimed to evaluate, for the first time in the literature, the influence of the extraction method and extract concentration on the morphological and biological properties (antioxidant and antibacterial activity) of silver nanoparticles phytosynthesized using *Echinacea pupurea* L. extracts. The obtained results revealed that the use of the low-concentration *Echinacea* hydro-alcoholic extract obtained via classical temperature extraction led to the development of nanoparticles with the smallest dimensions (less than 10 nm), compared with the use of extracts obtained with higher concentrations and the extract obtained via the microwave method. The developed nanomaterials exhibited enhanced antioxidant effects (determined via the DPPH assay) and antimicrobial properties (against *Escherichia coli* and *Candida albicans*), compared with the parent extracts.

## 1. Introduction

Nowadays, finding new solutions to replace the use of toxic chemical substances is a priority for researchers. Natural extracts can be considered a viable alternative in various areas, due to their chemical composition, being rich in natural bioactive compounds. In the nanotechnology domain, specifically in the area of phyto-nanotechnology (the synthesis of nanomaterials using natural extracts) their use represents a great opportunity, as the spectrum of usable plants is enormous, ranging from aromatic and medicinal plants to plant waste from various agricultural sectors. The properties of the obtained nanomaterials show a strong correlation with their morphological characteristics (i.e., size, shape, etc.) which in turn can be tailored by controlling the extracts’ composition [1], and there is a continuous need to explore low-cost synthesis methods and to identify green capping agents to prevent aggregation [2]. Classical methods of obtaining nanomaterials are being replaced by green synthesis methods [3], as part of the current drive to reduce energy consumption and avoid the use of toxic and harmful reagents [4].

The plants of the genus *Echinacea*, belonging to the Asteraceae family, are used for various medical purposes due to their antibacterial, antiproliferative, inhibitory, and antioxidant properties [5], and they are also used for obtaining metallic nanoparticles [6].

Before the development of modern synthetic medicines, herbal treatments derived from several species of the indigenous genus *Echinacea* were used. The plant has oval leaves with jagged edges, with a dark green and white upper surface. The flowers of the plant are dark purple, reddish purple, or pink, with a spiky central cone. The central cone of the flower acquires a sharper appearance as the flower matures [7]. The traditional ethnomedicinal use of *Echinacea* was related both to the wide distribution of the plant and to its demonstrated effectiveness in a number of diseases [8,9]. *Echinacea purpurea* L. is a perennial medicinal plant of the Asteraceae family, native to America, and is the most widely cultivated medicinal plant of this species. This plant gained its reputation due to its medicinal and phytochemical compounds, being used in the treatment of skin conditions, chronic arthritis, intestinal pain, convulsions, and cancer [10], and at the same time representing a valuable source of antioxidants, which can be formulated for oral consumption [11]. Recently, an *Echinacea* ethanol extract encapsulated in chitosan-silica nanoparticles was proven to have a protective role against meniscal/ligamentous-injury-induced osteoarthritis in the obese rats [12]. Moreover, the nano-encapsulated materials acted as a pain ameliorator, at the same time reducing cartilage proteoglycan losses.

Analyses of the composition of *Echinacea* extracts revealed that chicoric acid was the dominant component (among the phenylpropanoids) [13], along with phenolic compounds such as flavonoids [14], tannins, alkaloids, starch, furochromones, and glycosides [15], all of them acting as reducing agents and stabilizing agents in the synthesis of nanoparticles [16].

Due to their non-toxic character, different types of materials based on natural compounds from *Echinacea* extracts have been developed, especially for medical applications. Extracts encapsulated in chitosan nanoparticles have been used for the treatment of diabetes [17], hematite nanoparticles have been developed for their anti-bacterial activity against different species of bacteria [18], gold nanoparticles for their antioxidant effects [19] or zinc oxide nanoparticles for their antibacterial effects [20]. Oxyhydroxide nanoparticles have also been developed using a mixture of rosemary and *Echinacea* phenolic extracts, and their cytotoxic effect on A549 human lung adenocarcinoma cells led to the conclusion that the developed materials demonstrated significant anticancer effects [21].

Gecer et al. [22] used the aqueous extract from the aerial parts of echinacea for the phytosynthesis of silver nanoparticles with an average size of 68.24 nm (determined via scanning electron microscopy) and the nanoparticles exhibited very promising antioxidant activity (as determined by DPPH, ABTS, and ferric reducing power assays).

Starting from these premises, we aimed to evaluate the influence of two important factors affecting the final morphological and biological properties of the phytosynthesized nanoparticles: the extraction method applied to the vegetal material and the extract concentration used in the process. To our knowledge, the present study represents the first work describing these aspects of the extraction process.

## 2. Materials and Methods

### 2.1. Vegetal Material

**Figure 1:**
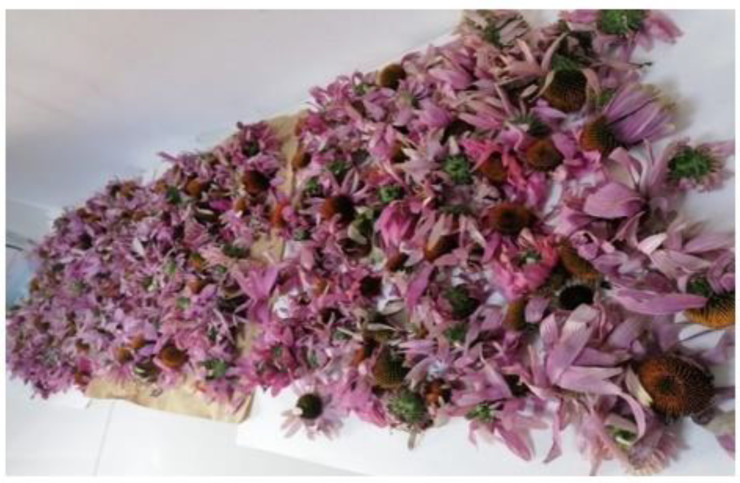
*E. purpurea* flowers used in the experiments.

The vegetal material (flowers of *Echinacea purpurea* L.) was collected in the Bucharest area from plants grown from certified seeds (Figure 1).

The drying of the plant material was performed away from the direct action of the sun, and drying was considered complete when a constant mass was reached. This method offers the maximum preservation of the active principles contained in the plant material, compared with other drying methods [23]. Once dried, the plant material was ground using a GRINDOMIX GM 200 mill (Retsch GmbH, Haan, Germany) and stored in the dark in airtight containers for further use.

### 2.2. Obtaining of Natural Extracts

To obtain the natural extracts, two extraction methods were selected, which, according to the literature data, ensured both the extraction of the active principles and the possibility of scaling up the processes, in consideration of future industrial applications [24].

In the classical temperature extraction method, the vegetable product, which was previously shredded, was subjected to classical temperature extraction using a hydroalcoholic mixture as a solvent, in a ratio of ethanol:water = 1:1, in a Memmert UN 110 oven (Memmert GmbH, Schwabach, Germany), with an extraction time of 3 h and a temperature of 70 °C. The obtained extract was encoded E_e_.

In the microwave-assisted extraction method, the crushed vegetable product, together with the hydroalcoholic solvent (ethanol:water = 1:1), was heated using microwave energy, using an Ethos Easy Advanced Microwave Digestion System (Milestone Srl, Sorisole, Italy), an extraction time of 30 min., an extraction temperature of 70 °C, and a microwave power of 800 W. The obtained extract was encoded E_mw_. For both extraction procedures, we maintained a vegetal material/solvent ratio of 1/12 (*w*/*v*). All the extracts obtained were filtered using filter paper.

After obtaining and filtering the extracts, they were reduced using a Laborota 4000 Heidolph rotary evaporator (Heidolph Instruments, Schwabach, Germany) to remove the volatile solvents. Finally, after removing more than 90% of the solvent, the extract was dried via lyophilization using a Christ LSC Alpha 2-4-LSC lyophilizer (Martin Christ Gefriertrocknungsanlagen GmbH, Osterode, Germany) in order to preserve the properties of the extract and to extend its shelf life. The extracts thus dried were stored in the freezer for further use. The powder thus obtained could be kept for a long time, in order to carry out all the experiments.

The ethanol used for the extraction procedures was of reagent quality (Chimreactiv, Bucharest, Romania), whereas the bidistilled water used for all experiments was obtained in the laboratory using a GFL 2102 water still (GFL, Burgwedel, Germany).

### 2.3. Characterization of Natural Extracts

One of the main factors affecting the morphology and dimensions of metal nanoparticles is the composition of the extract used. In order to elucidate the composition, phytochemical tests were selected (the total content of phenolic compounds, and the total flavonoids content), whereas high-precision liquid chromatography (HPLC) was applied to quantify the target compounds. All reagents were used as received, without further purification.

The total phenolics content was determined using a colorimetric method (the Folin–Ciocâlteu reagent method). This method involves the reduction of the Folin–Ciocâlteu reagent using phenolic compounds, with the formation of a blue complex. The diluted extract is mixed with the Folin–Ciocâlteu reagent (Merck KGaA, Darmstadt, Germany) and a precise quantity of sodium carbonate solution (Merck KGaA, Darmstadt, Germany) is added to each test tube. After 60 min, the optical density at 765 nm is measured using a Rigol Ultra 3660 UV-Vis spectrophotometer (Rigol Technologies, Beijing, China). The measurements were compared to a standard curve prepared with gallic acid solutions at different concentrations. The total phenolic content was expressed as milligrams of gallic acid equivalents (GAE) [25], using the following equation:

(1) $$C_{TP}=c\times \frac{V}{m}$$

where *C_TP_* is the total content of phenolic compounds (μg/g) in gallic acid equivalents (GAE), *c* is the concentration of gallic acid obtained from the calibration curve in μg/mL, *V* is the volume of the extract in mL, and *m* is the extract mass used (in grams).

Five determinations were performed, with the results being presented as the average of the determinations ± the standard error of the mean.

To determine the total flavonoid content, the extract was mixed with ethanol, aluminum chloride (10%, Merck KGaA, Darmstadt, Germany), potassium acetate (1M, Merck KGaA, Darmstadt, Germany), and bidistilled water. After 30 min of incubation at room temperature, the absorbance was measured at 415 nm. The measurements were compared to a standard curve prepared with rutin solutions in different concentrations. The total flavonoid content was expressed as milligrams of rutin equivalents (RE) [25], using the following equation:

(2) $$C_{TF}=c\times \frac{V}{m}$$

where *C_TF_* is the total flavonoid content (μg/g) in rutin equivalents (RE), *c* is the concentration of rutin obtained from the calibration curve in μg/mL, *V* is the extract volume in mL, and *m* is the extract mass used (in grams). Five determinations were performed, the results being presented as the average of the determinations ± the standard error of the mean.

High-performance liquid chromatography with a diode-array detector (HPLC-DAD, Rigol Technologies Inc., Beijing, China) was used for the identification and quantification of some selected components. In total, 10 µL extract was injected into the HPLC system and the compounds were chromatographed over a Kinetex EVO C18 column (150 × 4.6 mm, particle size = 5 µm) at 30 °C. The following combination of solvent mixtures was used: H_2_O + 0.1% TFA (solvent A) and ACN + 0.1% TFA (solvent B). The flow rate was 1 mL/min, the gradient elution ranged from 2% to 100% B, the time was set to 60 min, and detection was set at 300 nm. The stock solutions containing the reference compounds (catechin, epicatechin, coumaric acid, hyperoside, rutin, naringin, malvidin, genistein, gallic acid, caffeic acid, chlorogenic acid, and naringenin—all standards from Merck KGaA, Darmstadt, Germany) were prepared so that their concentration was 1000 µg/mL. For the calibration curves, concentrations between 10 and 400 µg/mL were used.

For these experiments, the lyophilized extracts were diluted in bidistilled water at a concentration of 5 mg/mL.

### 2.4. Phytosynthesis and Characterization of Silver Nanoparticles

For the development of phytosynthesized silver nanoparticles, the previously prepared dry extracts were used, redissolved in bidistilled water at various concentrations (5, 2.5, 2, 1.25, and 1 mg/mL, respectively) and mixed with the 10^−3^ M silver nitrate solution (Chimreactiv, Romania) in a 1:1 extract/metallic salt ratio. The extracts, according to the principles of phytosynthesis, play the role of a reducing and stabilizing agent for the formed nanoparticles [26]. The phytosynthesis process was initiated immediately after mixing the two components, and the formation of nanoparticles could be visually monitored due to the appearance of the specific ruby-red color of silver nanoparticles.

The encoding of the obtained samples is presented in Table 1.

The (phyto)synthesis of silver nanoparticles can be successfully observed using UV-Vis spectrometry, with silver nanoparticles exhibiting absorption maxima in the 400–550 nm region. Using this method, primary evaluations can be made in regard to both the sizes of the particles obtained and their stability [24]. The stability of the phytosynthesized AgNPs was observed over a period of several months. For the determinations, a Rigol Ultra 3660 UV-Vis spectrometer (Rigol Technologies Inc., Beijing, China, optical resolution 0.5 nm) was used, in the wavelength range of 375–550 nm.

To determine the hydrodynamic diameter of the nanoparticles by means of the DLS technique, five/ten determinations were made for each sample. The physicochemical characterization of the prepared nanomaterials is an important factor in the analysis of biological activities using radiation scattering techniques [27]. Nanoparticle dispersions were analyzed using a Zetasizer Nano ZS ZEN 3600 instrument (Malvern Instruments, Malvern, UK) capable of determining particle sizes in the range of 0.6–6000 nm.

X-ray diffraction (XRD) is a popular analytical technique that can be used for the analysis of both molecular and crystal structures, as well as determination of particle sizes, among other applications [28].

X-ray diffraction (XRD) analyses were performed using a 9 kW Rigaku SmartLab diffractometer (Rigaku Corp., Tokyo, Japan, operated at 45 kV and 200 mA, CuKα radiation—1.54059 Å), in scanning mode 2*θ*/*θ*, between 7° and 90° (2*θ*). Components were identified via comparisons with ICDD data.

Crystallite size was determined using the Debye–Scherrer equation:

(3) $$Dp=\frac{\left(K\times \lambda \right)}{\left(\beta \times \cos\theta \right)}$$

where *Dp* is the average size of the crystallites, *K* is the Scherrer constant (for cubic structures, *K* = 0.94), β represents the width at half-height of the diffraction maximum, *θ* is the Bragg angle, and *λ* is the wavelength (1.54059 Å in our case).

Transmission electron microscopy is a very useful, frequently used, and important technique for the characterization of nanomaterials, used to obtain quantitative measures of particle and/or grain size, size distribution, and morphology [29]. Transmission electron microscopy images were recorded using a Tecnai G2 F20 TWIN Cryo-TEM (FEI Company, Hillsboro, OR, USA) system, at an accelerating voltage of 300 kV and a resolution of 1 Å.

### 2.5. Evaluation of Antioxidant Properties

The antioxidant activity of the extracts and silver nanoparticles was determined using the DPPH assay. The DPPH test involves mixing the sample with a DPPH solution (Sigma Aldrich, St. Louis, MO, USA). The solutions were incubated for 30 min and subsequently the absorbance of the solutions was determined at 517 nm using a UV-Vis spectrophotometer [29]. The antioxidant activity (AA%) was calculated using the formula

(4) $$\mathrm{AA}\%=\frac{\left({A\;}_{\mathrm{blank}}-{A\;}_{\mathrm{sample}}\right)}{{A\;}_{\mathrm{blank}}}\times 100$$

where A_blank_ is the absorbance of the DPPH solution without the sample and A_sample_ is the absorbance of the extract mixed with the DPPH solution at 0.02 mg/mL.

Five determinations were performed, with the results presented as the average of the determinations ± the standard error of the mean.

### 2.6. Evaluation of Antimicrobial Properties

The antimicrobial properties were evaluated against two microorganisms, selected due to their pathogenicity.

*Candida albicans* is responsible for an infection called candidiasis, which can affect people of any age, but more often affects children or the elderly. The mortality rate for invasive candidiasis is relatively high, with an estimated percentage of 20–40% in infections in children, especially in premature babies. Likewise, mortality is high in the case of the elderly, especially when associated with other comorbidities [30]. One of the drugs used to combat *Candida albicans* yeast is miconazole, for which it was used as a positive control in this study (miconazole nitrate). In the present study, the strain *Candida albicans* ATCC 64548 was used for antimicrobial experiments.

The second pathogenic microorganism chosen for the antimicrobial testing of samples was *Escherichia coli* (*E. coli*) ATCC 8738 bacterium (Gram (−)). *Escherichia coli* is a bacterium that can cause serious infections. Some species produce a strong toxin that causes bloody diarrhea and, rarely, serious hematological problems and even kidney failure. The most common type of *E. coli* is *E. coli* O157:H7 [31]. Other types of *E. coli* can cause urinary tract infections or other infections, and death can occur in patients with various comorbidities. It is one of the bacteria that cause nosocomial infections in hospitals [32]. One of the antibiotics used to treat people infected with *Escherichia coli* is gentamicin [33,34,35]; for this reason, in this study gentamicin sulfate was chosen as a positive control.

Each experiment was carried out in triplicate, using distilled water as a negative control and, as a positive control, the commercial antimicrobials miconazole nitrate (Sigma Aldrich), at 30 µg/mL, and gentamicin sulfate (Sigma Aldrich) at 10 µg/mL.

*Escherichia coli* was grown in Luria Bertani agar (LBA, Miller, Boston, MA, USA), plates at 37 °C with a medium composition of 10 g/L casein enzymic hydrolysate, 5 g/L yeast extract, 10 g/L sodium chloride, and 15 g/L agar and *Candida albicans* was grown on Malt Extract Agar (MEA, Merck) with a culture medium composition of 17 g/L malt extract and 20 g/L agar. The methodology used for the agar well diffusion assay for antibiotic susceptibility has been described previously [29]. Briefly, sterile LBA and MEA Petri dishes were prepared by pouring the sterilized culture media into plates. One milliliter of microorganisms was spread on the agar plate’s surface and with a sterile borer, with which we made wells (6 mm in diameter). Fifty microliters of the tested samples were added into every well and the plates were placed in the incubator (Laboshake Gerhardt incubator) at 37 °C for 24–48 h.

According to the methodology described by Ponce et al. [36], the sensitivity of the microorganisms was established via measurements of their zone of inhibition (ZOI) diameters.

**Scheme 1:**
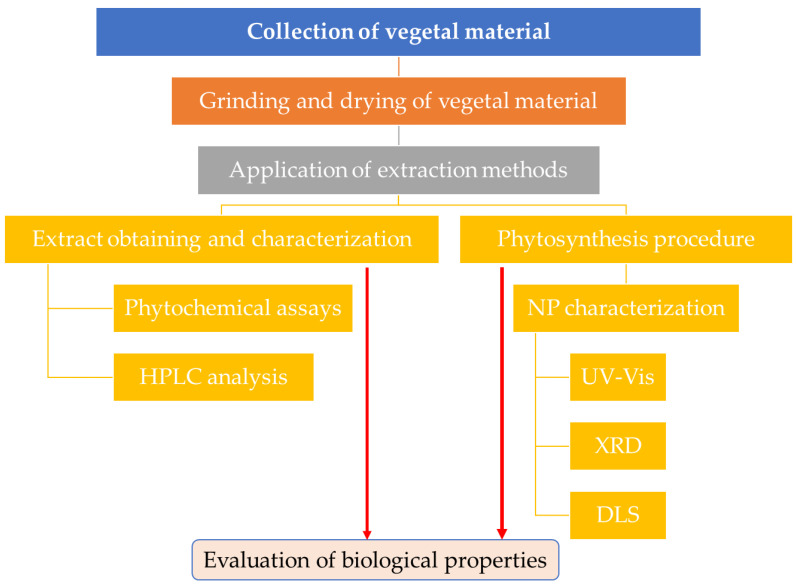
Flowchart of the proposed methodology.

A flowchart of the methodology applied in this study is presented in Scheme 1.

### 2.7. Statistical Analysis and Data Representation

The determinations were carried out through multiple parallel determinations (as mentioned for each method) and the data obtained were analyzed for statistical significance using analysis of variance (one-way ANOVA) and Tukey’s test to determine significant differences between means. Significant differences were set at *p* ≤ 0.05. The results shown are means ± standard error of the mean (SE) of independent determinations.

Graphical representations were constructed using OriginPro 2018 Data Analysis and Graphing Software (OriginLab Corporation, Northampton, MA, USA).

## 3. Results

### 3.1. Extract Characterization

The total phenolic and flavonoid content (calculated according Equations (1) and (2)), as well as the HPLC results, are presented in Table 2.

### 3.2. Characterization of Nanoparticles

**Figure 2:**
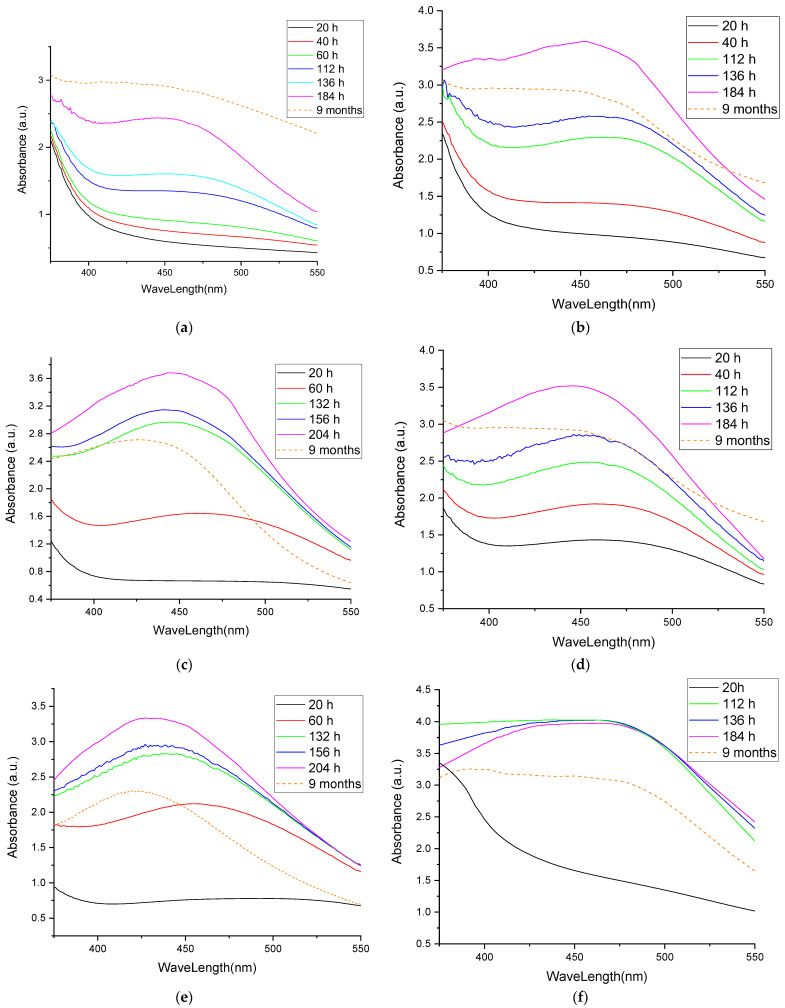

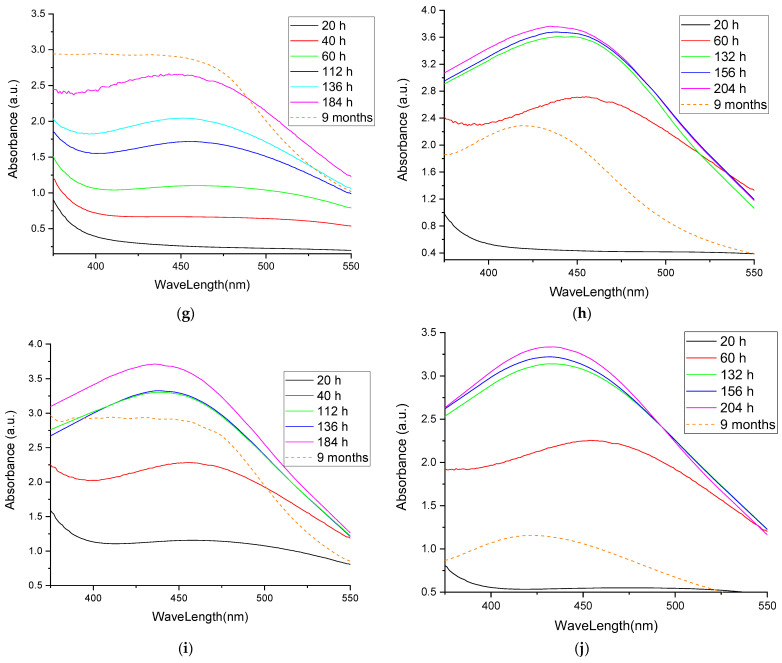
UV-Vis spectra of the obtained nanoparticle solutions: (**a**) sample EE5; (**b**) sample EE2.5; (**c**) sample EE2; (**d**) sample EE1.25; (**e**) sample EE1; (**f**) sample EM5; (**g**) sample EM2.5; (**h**) sample EM2; (**i**) sample EM1.25; (**j**) sample EM1.

As previously stated, the formation of silver nanoparticles can be followed using UV-Vis spectrometry. Figure 2 presents the UV-Vis spectra of the obtained nanoparticles, whereas the data obtained for the analyzed samples are presented in Table 3.

**Figure 3:**
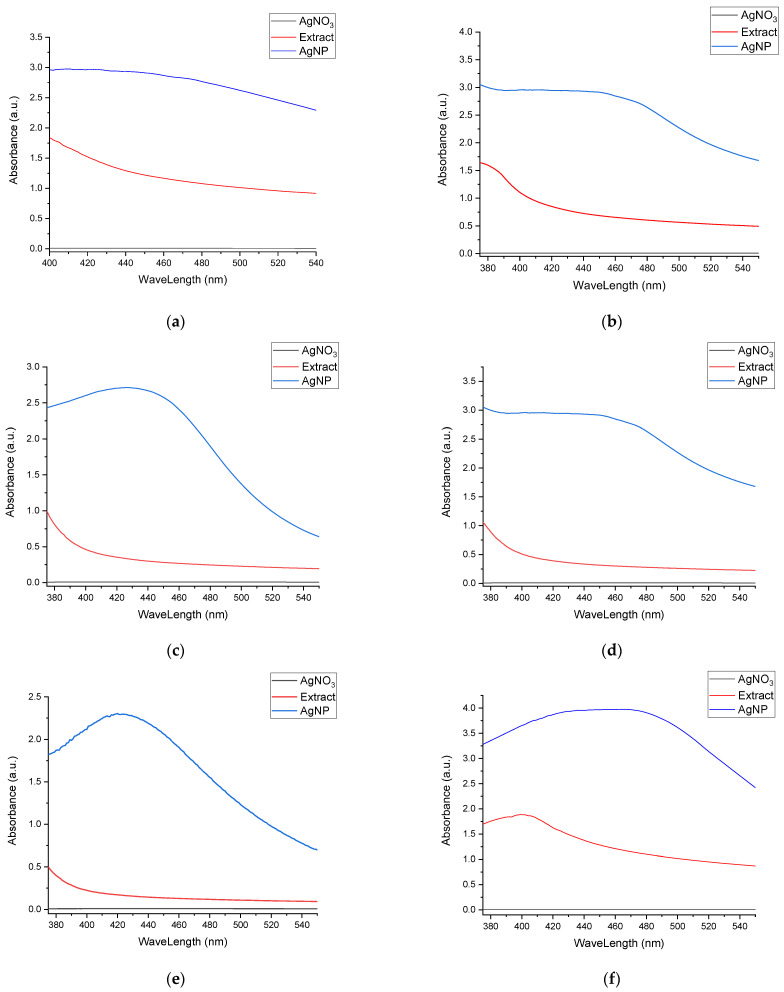

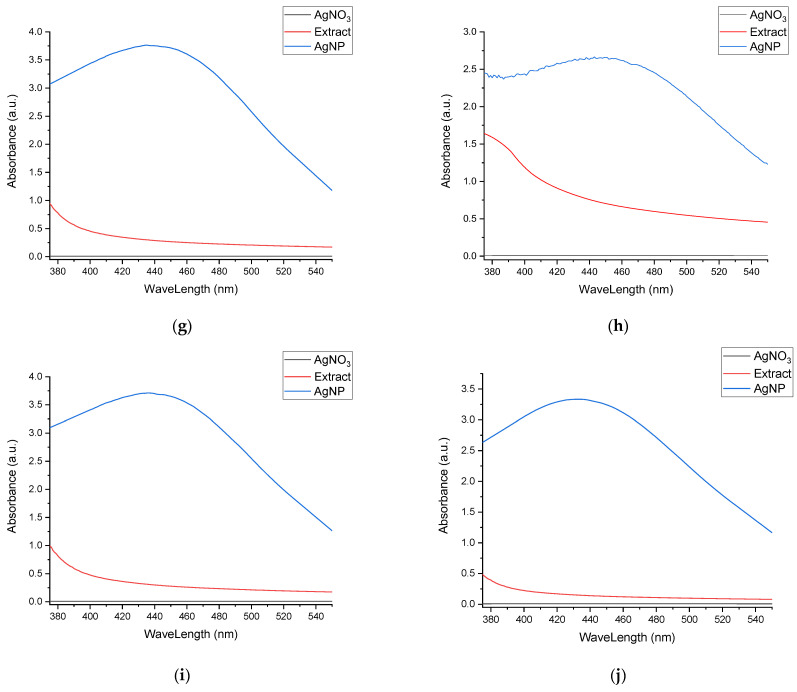
Confirmation of phytosynthesis of silver nanoparticles by the comparison of nanoparticles’ UV-Vis spectra with the corresponding extracts and AgNO_3_ solution: (**a**) sample EE5; (**b**) sample EE2.5; (**c**) sample EE2; (**d**) sample EE1.25; (**e**) sample EE1; (**f**) sample EM5; (**g**) sample EM2.5; (**h**) sample EM2; (**i**) sample EM1.25; (**j**) sample EM1.

In Figure 3, the superimposed UV-Vis spectra for the nanoparticle formulations at the end of the stability evaluation period are presented, compared to the corresponding extract and the silver nitrate solution, thus confirming the phytosynthesis of silver nanoparticles.

**Figure 4:**
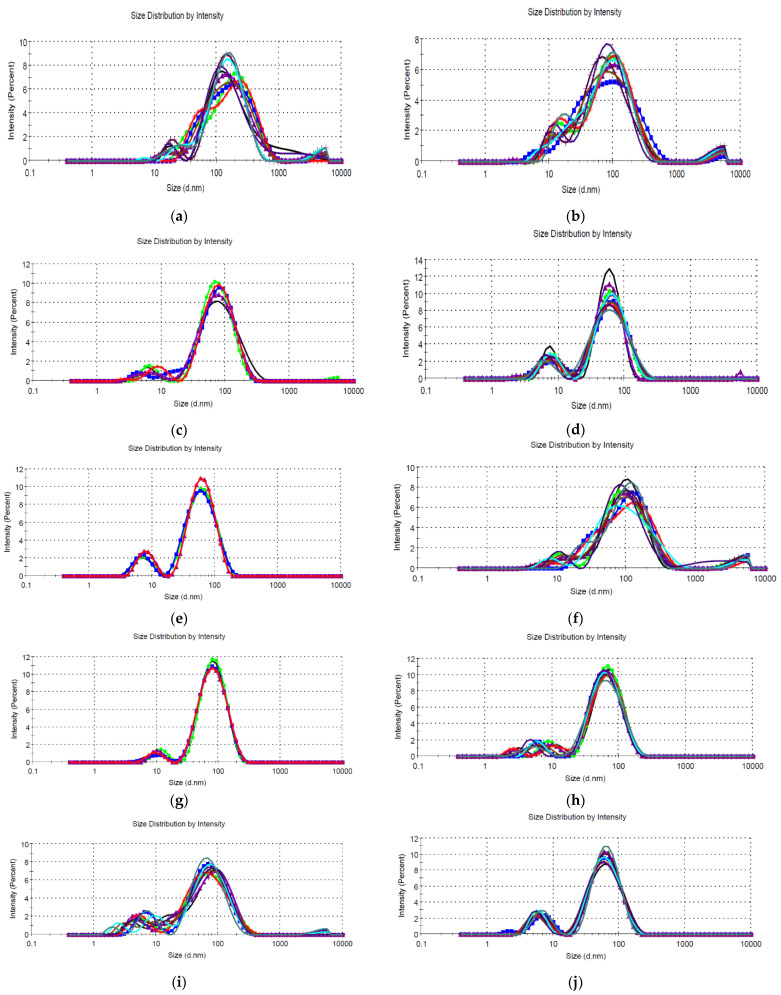
Size distribution by intensity, determined via the DLS technique, for (**a**) sample EM5; (**b**) sample EM2.5; (**c**) sample EM2; (**d**) sample EM1.25; (**e**) sample EM1; (**f**) sample EE5; (**g**) sample EE2.5; (**h**) sample EE2; (**i**) sample EE1.25; and (**j**) sample EE1.

To determine the sizes of the nanoparticles using the DLS technique, five/ten determinations were made for each sample (depending on the complexity of the sample) directly on the nanoparticle solution, without any dilution or treatment. The results are presented in Figure 4 and Table 4, respectively.

**Figure 5:**
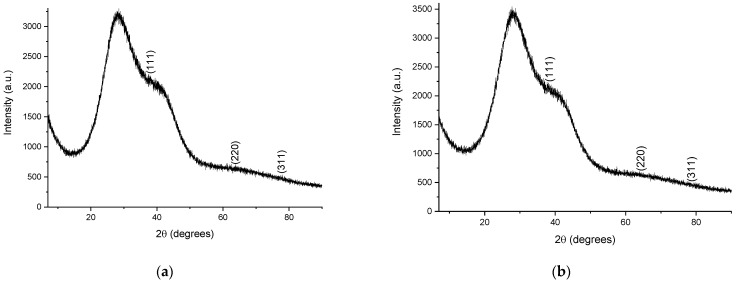

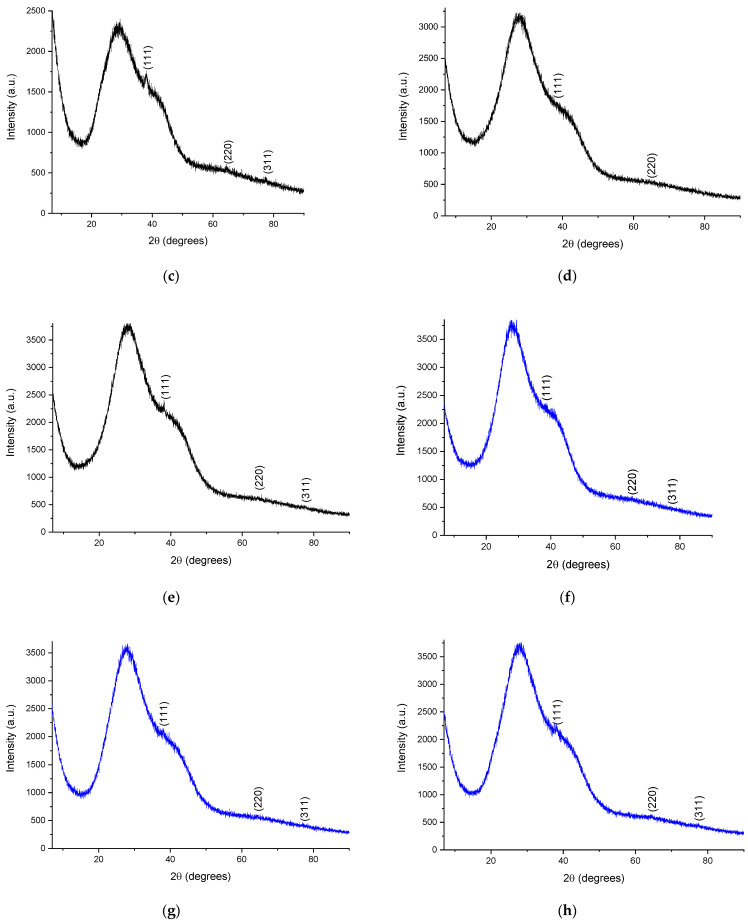

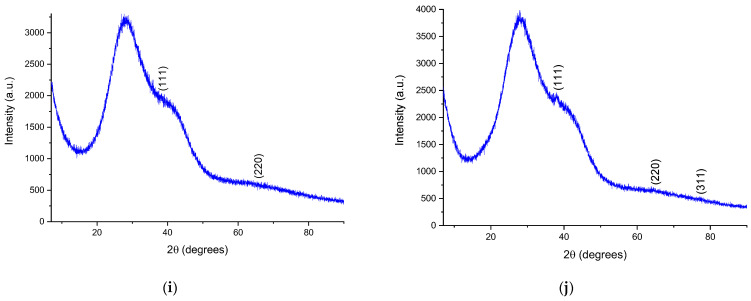
Diffractograms of the phytosynthesized nanoparticles: (**a**) sample EE5; (**b**) sample EE2.5; (**c**) sample EE2; (**d**) sample EE1.25; (**e**) sample EE1; (**f**) sample EM5; (**g**) sample EM2.5; (**h**) sample EM2; (**i**) sample EM1.25; (**j**) sample EM1; Miller indices are presented on each image.

To confirm the crystalline nature of the samples, the phytosynthesized nanoparticles were analyzed via X-ray diffraction. The diffractograms of the analyzed samples are presented in Figure 5, while the calculated crystallite dimensions are presented in Table 5. In order to analyze them using the XRD method, the samples containing the nanoparticle suspension were centrifuged using a DLAB DM0408 laboratory centrifuge, at 4000 rpm, for two hours. The precipitate thus obtained was deposited on the glass support for analysis.

**Figure 6:**
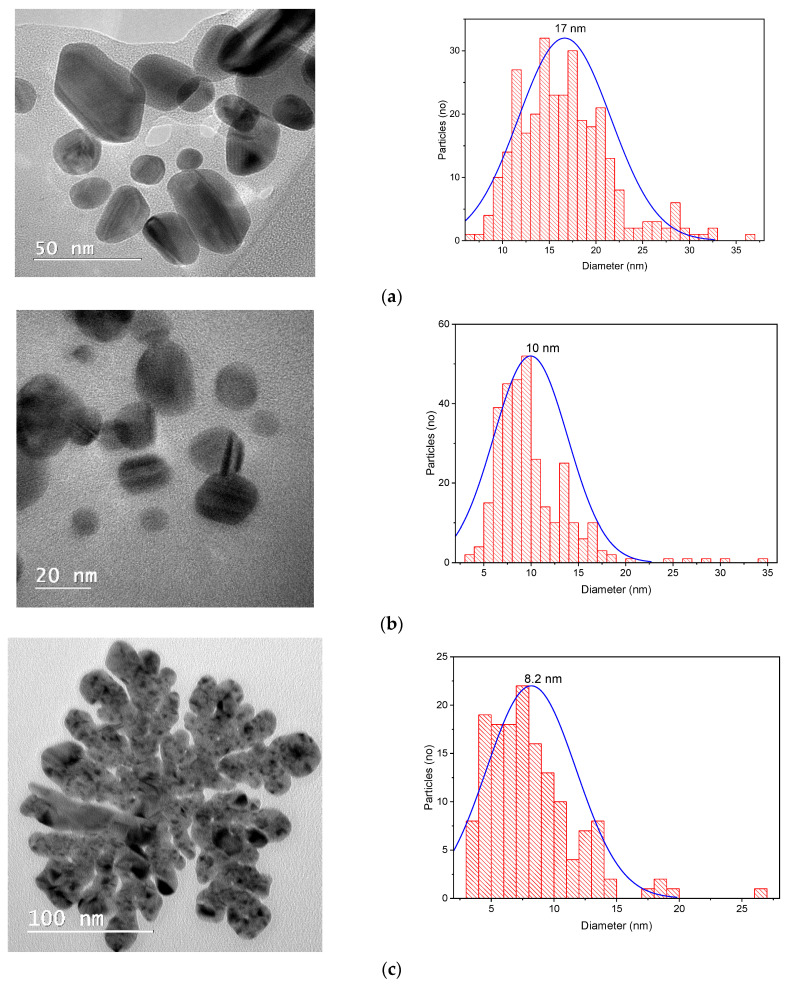

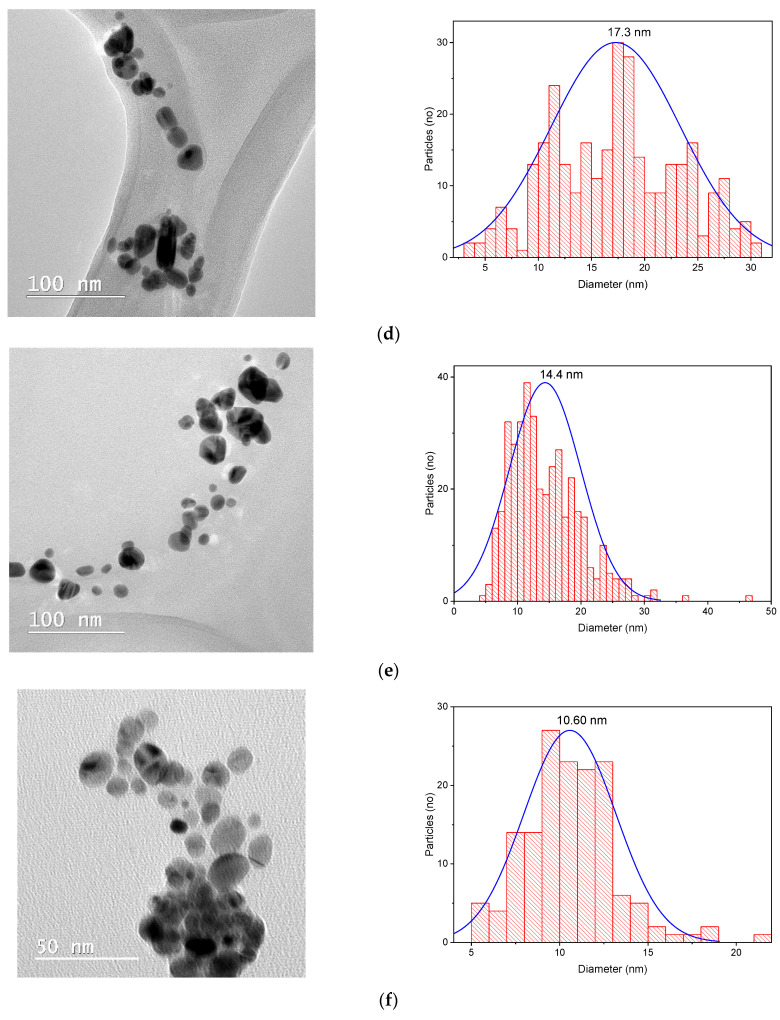
TEM images and size distributions obtained for the phytosynthesized nanoparticles: (**a**) sample EE5; (**b**) sample EE2; (**c**) sample EE1; (**d**) sample EM5; (**e**) sample EM2; (**f**) sample EM1.

Considering all the results obtained, three samples were selected for each set of phytosynthesized nanoparticles (the ones obtained using extracts with the maximum concentration—5 mg/mL, an average concentration—2 mg/mL, and the lowest concentration used—1 mg/mL) for the evaluation of the nanoparticle morphologies. For the analysis, a drop of extract containing the nanoparticle dispersion was diluted at a ratio of 1:10 (*v*/*v*), ultrasonated, and subsequently placed in the center of a copper grid, then dried and subjected to microscopic analysis. The TEM results are shown in Figure 6. The nanoparticle size distribution was examined via direct measurements of nanoparticles from TEM images (over 150 measurements) using ImageJ image analysis software (v. 1.53s, National Institutes of Health, Bethesda, MD, USA).

### 3.3. Evaluation of Antioxidant and Antimicrobial Properties

**Figure 7:**
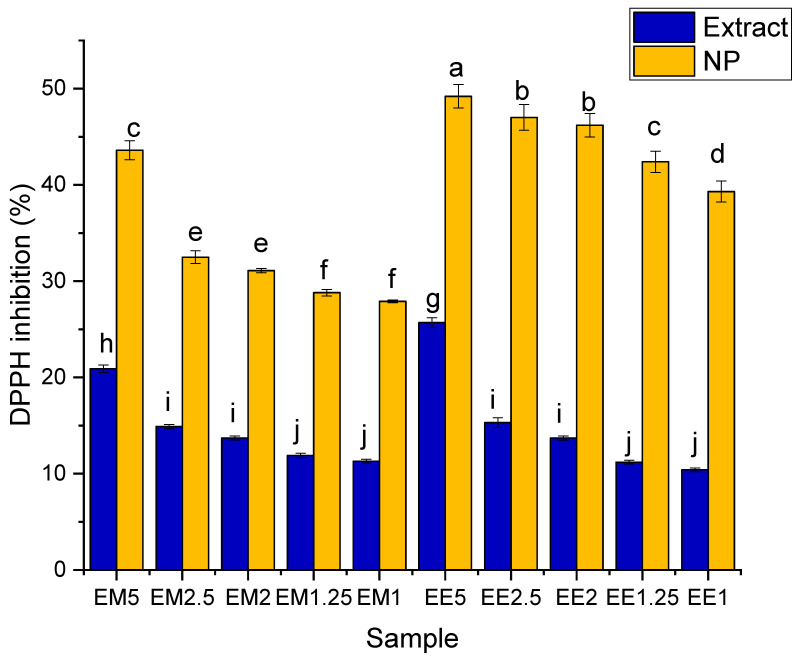
Antioxidant activity (expressed as DPPH inhibition—%) for the phytosynthesized nanoparticles and corresponding extracts. Values represent the mean ± SE, *n* = 5 per treatment group; values without a common lowercase letter differ statistically (*p* < 0.05) as analyzed via one-way ANOVA and the TUKEY test.

The results regarding the determination of antioxidant activity (obtained using Equation (4)) are presented in Figure 7.

**Figure 8:**
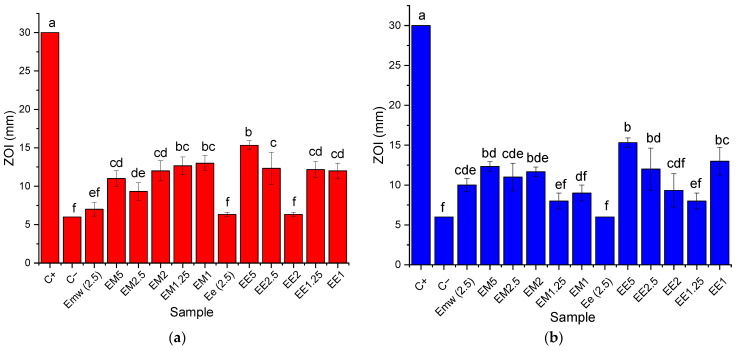
Antimicrobial activity of the tested samples against (**a**) *Candida albicans* ATCC 64548; (**b**) *Escherichia coli* ATCC 8738. C+—positive control (as described in Section 2); C−—negative control (water); E_mw_ (2.5)—microwave-assisted extract at 2.5 mg/mL; E_e_ (2.5)—classical temperature extract at 2.5 mg/mL. Values represent the mean ± SE, *n* = 3 per treatment group; values without a common lowercase letter differ statistically (*p* < 0.05) as analyzed via one-way ANOVA and the TUKEY test.

The results of the evaluation of antimicrobial properties are presented in Figure 8.

## 4. Discussion

### 4.1. Main Findings

The results of the phytochemical assays and HPLC analysis (Table 2) revealed the influence of the extraction method on the extract’s composition. Thus, the content of total phenolic compounds (expressed as μg GAE/g extract) was significantly higher for the classical temperature extraction method, whereas the microwave-assisted extraction method led to the extraction of a significantly higher content of flavonoids (expressed as mg RE/g extract). The data in the literature data present comparable results, with TPC values of approx. 400 mg chlorogenic acid equivalents/g extract [37] and approx. 350 mg GAE/100 g extract for the ethanolic extract [38], as presented by other authors.

The difference between the results presented in the current study and the literature data [39] can be explained, most likely, by the influence of environmental factors and cultivar quality, leading us to the conclusion that *E. purpurea* samples grown under native conditions exhibit a lower intake of phenolic compounds. At the same time, the selected extraction methods (using milder conditions and “greener” solvents) could also negatively influence the total phenolic content.

In terms of total flavonoids, other studies identified the presence of approx. 11 mg catechin equivalents/100 g ethanolic extracts [38] and between 10 and 33 quercitin equivalents/g dry weight for different parts of *E. purpurea* extracts grown in supplemented soil [40]. The total flavonoid content observed in this study was higher than the flavonoid contents reported by other authors [39], leading to the conclusion that the selected methods positively influenced the total flavonoid content.

Among the components identified via HPLC (some of which were quantified in the *E. purpurea* extracts for the first time in this study), the highest contents were recorded for hyperoside, malvidin, catechin, and epicatechin, with most compounds being identified in higher contents in the extract obtained using the microwave-assisted method. The contents of particular compounds are highly variable among studies, i.e., other authors have reported values of less than 4 mg/500 g powder for catechins for pharmaceutical forms of *Echinacea* [41], whereas rutin (previously identified in echinacea extracts [17]) has been rarely quantified.

The characterization of the extracts, although not the main goal of the present study, represents an important parameter for defining one of the factors influencing the physico-chemical properties of the phytosynthesized nanoparticles. The elucidation of the extracts’ qualitative composition, as well as the identification of some of the important components of the obtained extracts, represent important aspects of research which will be useful for future studies in the same area.

The UV-Vis evaluation of the phytosynthesis process (Figure 2a–e) revealed that, in the case of echinacea extracts obtained via classical temperature extraction, with the decrease in the concentration of the extract, the formation of nanoparticles was faster, and the formation of the characteristic maximum could be observed from the first observation times. Thus, in the case of EE5, the characteristic maximum appeared only after 112 h of reaction, in the case of EE2.5 it was faintly visible at 40 h and well-defined at 112 h, and in the case of EE2 it was more clearly defined at 60 h, becoming very clearly defined for the samples EE1.25 and EE1 at 40 and 60 h, respectively. In addition, in the case of extracts with lower concentrations, a smaller shift in the specific maxima could be observed, which implies a smaller variation in the particle sizes. The smallest apparent particle sizes after 9 months were recorded for sample EE1. A particular case of note was the determination made at 184 h for sample EE2.5—the appearance of a secondary maximum at 392 nm was probably associated with the presence of nanoparticles with low dimensions (less than 10 nm).

Compared to the results regarding phytosynthesis based on extracts obtained using the classical method, a similar trend was observed in the case of extracts obtained using microwaves. In Figure 2f–j it can be observed that, in the case of *Echinacea* extracts obtained by means of microwave-assisted method, with a decrease in the extract concentration, the maximum characteristics of silver nanoparticles were more clearly defined. Furthermore, in the case of the lowest extract concentration used (sample EM1), the smallest shift in the characteristic maximum was observed for the determinations at 132, 156, and 204 h, and in the case of comparing these maxima with the one determined in the case of the nanoparticle stability assessment undertaken after 9 months. In the case of nanoparticles phytosynthesized using *Echinacea* extracts obtained using microwave extraction, according to the evaluation of the size of the nanoparticles, the lowest values after 9 months were recorded for samples EM2 and EM1.

The most probable explanation for these results is the sudden reduction of a large amount of silver to the Ag^0^ form in the case of more concentrated extracts, which may be correlated with the extract’s phytoconstituents’ weak capacity to stabilize them. Furthermore, in the case of less concentrated extracts, the particle size was apparently smaller, but it should be noted that the position of the maximum is also influenced to a small extent by the color of the extract, a contribution that decreases with dilution. The phytosynthesis is further confirmed when comparing the spectra registered with the ones of the extracts, respectively the silver salt (Figure 3).

The DLS measurements performed directly on the nanoparticle-containing solutions (Figure 4a–j) revealed the presence of the nanoparticles. Although some samples contained large aggregates (Table 3), their proportion was minor, especially in the case of samples with low extract concentrations (≤2%). In general, nanoparticle sizes determined via the DLS method exhibited hydrodynamic diameters that were often much larger than the actual nanoparticle diameters [42]. However, for all phytosynthesized nanoparticles, the observations undertaken via the UV-Vis spectrometry analysis were confirmed, namely, the obtaining of particles with lower sizes when using solutions with lower extract concentrations, in accordance with the literature data [42].

The polydispersity index (PdI) is dimensionless and scaled so that values < 0.05 represent the characteristics of a monodisperse standard sample. In addition, values greater than 0.7 signify a very broad distribution of particle sizes, in which case the DLS method is not suitable for analysis, as the algorithms associated with the method work in the range of PdI values of 0.05–0.7. In practice, values of PdI < 0.5 lead to the conclusion that a sample can be considered monodisperse, and higher values are associated with samples with a moderate polydispersity [43].

X-ray diffraction data for most of the samples displayed three diffraction maxima corresponding to the diffraction planes: (111)—approx. 38°, (220)—approx. 64°, and (311)—approx. 77°, respectively, confirming the synthesis of Ag nanoparticles in a cubic crystalline system, identified based on ICDD entry 01-087-0719.

The XRD data confirmed the results obtained previously, in the sense that the smallest crystallite sizes (Table 5) were obtained when using lower concentrations of the extract for phytosynthesis. The crystallite size, determined using the Scherrer equation, although not a true particle dimension determination (given the fact that, on one hand, the crystallite size determination is influenced by a series of factors, such as strain, equipment errors, residual stress, etc., and, on the other hand, the particles can be formed by multiple crystals), can also provide useful information on the NPs’ morphological characteristics. Moreover, in our particular case, the crystallite sizes followed the same trends observed via the UV-Vis and DLS measurements, namely, that the use of a lower concentration of extracts led to NPs with lower crystallite sizes, whereas the classical extraction method resulted in lower dimensions, compared with the microwave extraction method.

The most reliable technique when evaluating the dimensions of nanoparticles is electron microscopy (particularly, transmission electron microscopy). TEM analyses confirmed (Figure 6) the synthesis of nanoparticles with spherical or quasi-spherical morphologies (although other types of morphologies were also encountered, such as triangular, hexagonal, and ellipsoidal), with sizes below 30 nm in general, and with a tendency to form nanoparticles with smaller sizes when using lower-concentration extract solutions. Furthermore, when using the extracts obtained via the classical method, nanoparticles with lower sizes were usually obtained, although the differences were relatively small. However, in these cases, the morphology of the nanoparticles tended to be more irregular, compared with those obtained with extracts obtained through the microwave-assisted procedure.

There were large differences in the results obtained using different methods for the evaluation of the nanoparticles (in terms of absolute dimensions); however, all the analyses performed supported our general conclusions that a smaller concentration of extracts led to smaller NPs, whereas the use of extracts obtained via the classical temperature extraction method also led to smaller NPs, compared with the microwave extraction method. The differences recorded between the results of different methods of analysis can be explained by the fact that XRD provides the crystallite size (not the particle dimensions), whereas UV-Vis provides a more qualitative evaluation of the NP dimensions, rather than a rigorous analytical determination. The differences recorded between the DLS and TEM analyses can be explained by several factors. First of all, as previously stated [42], DLS expresses the hydrodynamic diameter of the nanoparticles, which, in our case—with the phytocomponents acting as capping agents—would be expected to be much larger than the nanoparticle diameter. Another important factor is the sample preparation method used for the analyses. For the TEM analysis, in the sample preparation stage, we avoided the agglomeration of the sample as much as possible, which led to more accurate results. Other studies in the literature have also recorded large differences when comparing DLS and TEM data (up to five time higher for DLS, especially when measuring the size distribution by intensity), and the errors recorded for DLS measurements increased for non-monomodal samples and for samples presenting very small nanoparticles (below 15 nm) or agglomerates [44,45]. As such, in the particular case of phytosynthesized nanoparticles, of which the hydrodynamic diameter was greatly influenced by the presence of capping phytocomponents, and in which the nanoparticle solutions consisted of nanoparticles of differing dimensions (which could also form larger aggregates), the method which led to the most reliable results was electron microscopy.

When comparing our results with the literature data, it can be noted that using the selected extracts, we obtained nanoparticles with dimensions comparable with those obtained using *Raphanus sativus* L. waste extracts [29], *Asplenium scolopendrium* L. leaves [46], *Aconitum toxicum* Reichenb. leaves [47], and *A. toxicum* rhizomes [48].

To our knowledge, the literature contains only one study regarding the phytosynthesis of silver nanoparticles using *Echinacea* flower extracts. Although presenting major differences in terms of both the extraction method and phytosynthesis procedure, which means the results are hardly comparable, it is worth mentioning that the authors of the cited study obtained much larger nanoparticles (over 60 nm in diameter), although they had a more uniform shape [22]. The higher dimensions of the nanoparticles recorded by these authors can be foreseen even based on the position of the characteristic plasmon resonance peak (481 nm in the cited work, compared to less than 442 nm in our case; see Table 3). The lack of literature data regarding the phytosynthesis of silver nanoparticles using *Echinacea* extracts underlines the need for further studies in this area, considering the wide spread and use of the plant.

Based on the results of the evaluation of biological properties, a significant increase in antioxidant activity could be observed for precursor extracts, which was directly proportional to the concentration. This variation was also preserved in the case of samples containing nanoparticles. After phytosynthesis, a statistically significant variation in the antioxidant activity was observed when comparing the values obtained in the case of phytosynthesized nanoparticles with those obtained for the “parent” extract.

The antioxidant properties (Figure 7) could be correlated with the values obtained for total phenolic compounds. Thus, as significantly higher values in terms of TPC were recorded for E_e_, the antioxidant activity was also significantly higher for EE5 compared with EM5. For the rest of the extract concentrations, there were no significant differences between the types of extracts at similar concentrations. The TF content, due to the minor (although statistically different) differences recorded, seems to have had a minor influence on the total antioxidant activity. A major difference was observed for the phytosynthesized nanoparticles, for which most of NPs obtained using classical extraction presented significantly higher antioxidant activity than those obtained using microwave-assisted extraction (except for sample EM5). This phenomenon can be explained by the synergistic action of several types of compounds in relation to the antioxidant activity, namely, by the greater efficiency of the classical extraction method in the extraction of several types of compounds with antioxidant activity. The high increase in the antioxidant activity observed for the EE series could also be explained by the involvement of secondary metabolites in the capping of NPs, which had a greater influence on the final antioxidant activity.

As can be observed in Figure 8, the phytosynthesized nanoparticles possessed good antimicrobial properties. In order to remove the influence of the extract on the final results, the antimicrobial properties of 2.5 mg/mL extracts (equal to the concentration of the extracts found in samples EE5 and EM5, respectively) were also evaluated. As a general remark, the phytosynthesized nanoparticles’ antimicrobial properties (presented as the zone of inhibition of a targeted line in mm) started from relatively high values corresponding to the highest concentration of the extract and slowly decreased, reaching a plateau (EM2.5 and EE2 for *Candida albicans* ATCC 64548 and EM1.25 and EE1.25 for *Escherichia coli* ATCC 8738) after which a sudden increase was observed. Most likely, this effect was due to the appearance of nanoparticles with small dimensions (as previously shown, with the smallest nanoparticles being recorded for the EX1 samples, where X = E or M). As such, it can be concluded that the nanoparticles’ dimensions strongly influenced the final antimicrobial properties. This dependance of the antimicrobial properties on the NPs’ dimensions was also recorded by our group for other phytosynthesized nanoparticles [46]. This confirms that when considering the antimicrobial properties of nanoparticles obtained using similar recipes, their dimensions should be carefully controlled. At the same time, the results suggest that in future experiments, low-concentration extracts could be used for the phytosynthesis process, thus providing a more economical recipe for developing active materials based on phytosynthesized nanoparticles.

### 4.2. Implications

The literature survey performed before the start of the experiments revealed a surprisingly low number of studies dealing with *Echinacea* in general, and with the implications of this species in nanotechnology in particular. Our findings highlight the potential application of *Echinacea* extracts in the development of phytosynthesized nanomaterials. The area of phyto-nanotechnology, due to the synergistic effects of the nanomaterials and phytoconstituents, could represent a very important resource in biological applications.

Among the factors that influence the morphology and dimensions of the nanoparticles, some of the ones related to the plant material have been addressed for the first time in this study, to our knowledge. Thus, the influence of the extraction method and of the natural extract concentration on the obtained materials and their applications was evaluated and discussed.

The complex chemical characterization of the extracts revealed significantly higher concentrations of total phenolic compounds for the classical temperature extraction method, whereas the microwave-assisted extraction method led to the extraction of significantly higher amounts of flavonoids. Among the components identified via HPLC (some quantified in the *E. purpurea* extracts for the first time in this study), the highest contents were recorded for hyperoside, malvidin, catechin, and epicatechin, with most compounds being identified in higher contents in the extract obtained via the microwave-assisted method. The chemical characterization of the synthesized silver nanoparticles showed them to have a cubic crystalline system, in the sense that the smallest crystallite sizes were obtained when using lower concentrations of the extract for phytosynthesis. The synthesis of nanoparticles with spherical or quasi-spherical morphologies (although other types of morphologies were also encountered, such as triangular, hexagonal, and ellipsoidal), with sizes below 30 nm in general, and with a tendency to form nanoparticles with smaller sizes when using lower-concentration extract solutions, was also confirmed.

The antioxidant properties could be correlated with the values obtained for total phenolic compounds. Thus, as significantly higher values in terms of TPC were recorded for Ee, the antioxidant activity was also significantly higher for EE5 compared with EM5. For the rest of the extract concentrations, there were no significant differences between the types of extracts at similar concentrations. The TF content, due to the minor (although statistically different) differences recorded, seems to have had a minor influence on the total antioxidant activity. A major difference was observed for the phytosynthesized nanoparticles, with most of NPs obtained using classical extraction method displaying significantly higher antioxidant activity than those obtained using microwave-assisted extraction (except for sample EM5). As a general remark, the phytosynthesized nanoparticles’ antimicrobial properties (presented as the zone of inhibition of a targeted line in mm) started from relatively high values corresponding to the highest concentration of the extract, and slowly decreased, reaching a plateau (EM2.5 and EE2 for *Candida albicans* ATCC 64548 and EM1.25 and EE1.25 for *Escherichia coli* ATCC 8738), after which a sudden increase was observed.

The obtained results support the initial hypothesis that the *Echinacea* extract can be successfully used for obtaining stable, biologically active silver nanoparticles. Moreover, the use of lower concentrations led to the phytosynthesis of nanoparticles with lower dimensions and with a superior antimicrobial effect.

Future studies should be focused on the evaluation of other factors involved in the phytosynthesis process, i.e., those related to the metal salt concentration used and the synthesis conditions.

By optimizing these different phytosynthesis process parameters, it will be possible to develop a scalable solution for practical applications, not only with greater biological effects, but also with lower economic costs.

## 5. Conclusions

The influence of the use of *E. purpurea* extracts (obtained via classical temperature extraction and microwave-assisted extraction methods) on the morphological (size and shape) and biological properties of the phytosynthesized silver nanoparticles (antioxidant and antibacterial effects against *Candida albicans* and *Escherichia coli*) was studied in the present paper.

The results support the application of the *Echinacea* hydro-alcoholic extract obtained via classical temperature extraction, used at low concentrations (1 mg dried extract/mL), to obtain spherical/quasi-spherical nanoparticles with average dimensions under 10 nm. 

The developed nanomaterials exhibited enhanced antioxidant effects (determined by the DPPH assay) and antimicrobial properties (against *E. coli* and *C. albicans*), compared with their parent extracts.

As a general conclusion, silver nanoparticles obtained with *Echinacea* extracts represent good candidates for further medical applications, with the process leading to the development of active materials even at low concentrations of the natural extracts.

## Figures and Tables

**Table 1:** Encoding of the phytosynthesized nanoparticle samples.

Extract Used	Extract Concentration (mg/mL)	NP Encoding
Classical temperature extract (E_e_)	5.00	EE5
	2.50	EE2.5
	2.00	EE2
	1.25	EE1.25
	1.00	EE1
Microwave-assisted obtained extract (E_mw_)	5.00	EM5
	2.50	EM2.5
	2.00	EM2
	1.25	EM1.25
	1.00	EM1

**Table 2:** The total content of phenolic compounds (TPC) and flavonoids (TF) in the analyzed extracts, according to the HPLC results ^1^.

Extract/Parameter	E_e_	E_mw_
TPC (μg GAE/g extract)	790.23 ± 12.51 ^a^	672.25 ± 14.83 ^b^
TF (mg RE/g extract)	71.54 ± 0.53 ^b^	72.82 ± 0.44 ^a^
Catechin (mg/g)	51.008 ± 1.551 ^b^	55.004 ± 1.752 ^a^
Epicatechin (mg/g)	25.059 ± 0.421 ^b^	26.728 ± 0.371 ^a^
Coumaric acid (mg/g)	0.013 ± 0.001 ^b^	0.029 ± 0.001 ^a^
Hyperoside (mg/g)	211.96 ± 4.547 ^b^	252.45 ± 4.318 ^a^
Rutin (mg/g)	0.228 ± 0.009 ^b^	0.623 ± 0.016 ^a^
Naringin (mg/g)	0.166 ± 0.004 ^b^	0.225 ± 0.01 ^a^
Malvidin (mg/g)	121.92 ± 1.221 ^a^	113.99 ± 1.891 ^b^
Genistein (mg/g)	0.273 ± 0.01 ^a^	0.152 ± 0.006 ^b^
Gallic acid	N.D.	N.D.
Caffeic acid	N.D.	N.D.
Chlorogenic acid	N.D.	N.D.
Naringenin	N.D.	N.D.

^1^ Values represent the means of five determinations (for TPC and TF) or three determinations (for HPLC results) ± SE; values in the same row without a common superscript letter differed statistically (*p* < 0.05) as analyzed by one-way ANOVA and the TUKEY test; N.D.—not detected.

**Table 3:** Evaluation of particle formation using *Echinacea purpurea* L. extracts by UV-Vis spectrometry as a function of reaction time (P_max_—characteristic peak maximum position, D_NP_—evaluation of nanoparticle sizes using the characteristic maximum position).

Time	E_e_ Samples										E_mw_ Samples									
	EE5		EE2.5		EE2		EE1.25		EE1		EM5		EM2.5		EM2		EM1.25		EM1	
	P_max_ (nm)	D_NP_ (nm)	P_max_ (nm)	D_NP_ (nm)	P_max_ (nm)	P_max_ (nm)	D_NP_ (nm)	P_max_ (nm)	D_NP_ (nm)	P_max_ (nm)	P_max_ (nm)	D_NP_ (nm)	P_max_ (nm)	D_NP_ (nm)	P_max_ (nm)	D_NP_ (nm)	P_max_ (nm)	D_NP_ (nm)	P_max_ (nm)	D_NP_ (nm)
20 h	-	-	481	78	460	68	460	67	485	79	482	77	-	-	-	-	459	66	474	76
40 h	471	75	475	76	-	-	458	66	-	-	470	73	470	73	-	-	455	64	-	-
60 h	470	73	-	-	459	66	-	-	456	64	-	-	455	64	454	63	-	-	454	63
112 h	446	57	461	67	-	-	453	62	-	-	455	64	453	62	-	-	439	49	-	-
132 h	-	-	-	-	444	55	-	-	440	50	-	-	-	-	441	51	-	-	433	45
136 h	444	55	459	66	-	-	450	60	-	-	455	64	448	58	-	-	437	48	-	-
156 h	-	-	-	-	442	53	-	-	435	47	-	-	-	-	439	49	-	-	431	42
184 h	442	54	451/392	61	-	-	444	55	-	-	455	64	443	54	-	-	435	47	-	-
204 h	-	-	-	-	443	54	-	-	428	42	-	-	-	-	437	48	-	-	430	41
9 months	423	40	439	49	423	40	442	53	420	33	448	58	432	44	420	33	434	47	421	35

**Table 4:** Results obtained for the measurement of the particle size via the DLS method. In the table: Px—maximum (peak) X, SPx—standard deviation associated with maximum X, %Px—% intensity Px, PdI—polydispersity index.

Sample	P_1_	SP_1_	% P_1_	P_2_	SP_2_	% P_2_	P_3_	SP_3_	% P_3_	Average	PdI	DLS Observations
EM5	347.5	594.8	94.8	18.27	3.945	5.2				116.9	0.457	Bimodal, reproducible
EM2.5	111.9	68.62	77.2	18.14	5.72	20.5	4397	922.6	2.2	49.53	0.532	Bimodal, reproducible, contains large aggregates
EM2	95.37	62.55	93.4	6.358	1.623	6.6				52.35	0.474	Bimodal, reproducible, polydisperse
EM1.25	62.12	21.94	84.2	7.617	1.675	15.8				37.38	0.455	Bimodal, reproducible
EM1	68.22	33.75	87.3	7.540	2.621	7.54				36.37	0.469	Monomodal, reproducible
EE5	109.7	58.14	84	14.77	5.385	9.4	4233	992.6	5.9	71.55	0.436	Monomodal, reproducible, polydisperse, contains large aggregates
EE2.5	86.55	38.26	94.8	9.506	3.142	5.2				64.64	0.285	Bimodal, reproducible, polydisperse
EE2	75.71	33.87	87.5	11.80	3.745	8.2	3.476	0.817	4.3	42.13	0.498	Bimodal, reproducible, polydisperse
EE1.25	96.86	59.40	78.9	14.22	4.471	19.5	4.243	1.139	1.6	36.70	0.585	Bimodal, reproducible, polydisperse
EE1	69.13	28.71	84.2	7.07	1.997	15.8				33.0	0.570	Bimodal, reproducible

**Table 5:** The positions of the diffraction maxima and the crystallite sizes of the analyzed samples, calculated according to Equation (3).

Sample	(111) Peak Position (Degrees)	(220) Peak Position (Degrees)	(311) Peak Position (Degrees)	FWHM (Degrees) ^1^	Crystallite Dimension (nm) ^1^
EE5	37.97	64.92	77.99	2.243	3.91
EE2.5	38.12	64.03	77.88	2.169	4.05
EE2	38.10	64.25	77.96	2.289	3.83
EE1.25	38.05	64.62	-	2.584	3.40
EE1	38.10	64.32	77.43	2.788	3.15
EM5	38.17	65.45	77.04	2.032	4.32
EM2.5	38.10	64.41	77.23	1.345	6.52
EM2	38.04	64.52	77.43	2.306	3.80
EM1.25	38.04	64.27	-	2.307	3.80
EM1	38.33	64.90	77.21	2.652	3.31

^1^ Data for the (111) diffraction plane.

## Data Availability

The data presented in this study are available on request from the corresponding authors.
